# Prevalence and demographic factors associated with MSD-related sick leave among dentists in Kuwait: a nationwide retrospective study

**DOI:** 10.1186/s12889-026-28153-y

**Published:** 2026-06-17

**Authors:** Saleh Alsarhan, Jagan Kumar Baskaradoss

**Affiliations:** 1 Department of Health Policy and Management, College of Public Health, Kuwait University, Sabah Al Salem University City, Kuwait City, 13060 Kuwait; 2 Department of Developmental and Preventive Sciences, College of Dentistry, Kuwait University, Sabah Al Salem University City, Kuwait City, 13060 Kuwait

**Keywords:** dentists, workplace, absenteeism, occupational health, musculoskeletal disorders, MSD-related sick leave, ergonomics, Kuwait

## Abstract

**Background:**

Musculoskeletal disorders (MSDs) are a major occupational health concern among dentists, contributing substantially to morbidity and work absenteeism. However, population-level evidence from Kuwait is limited. This study aimed to estimate the prevalence of MSD-related sick leave among dentists and to identify associated demographic factors.

**Methods:**

A nationwide retrospective observational study was conducted using administrative sick leave records from 1 January 2022 to 6 October 2023 across Ministry of Health (MOH) facilities in Kuwait. The analytic records included 2,735 dentists. MSD-related sick leave episodes were identified using an iteratively refined keyword-based algorithm incorporating diagnostic keywords, root terms, and bilingual terminology. The primary outcome was the occurrence of at least one MSD-related sick leave episode during the study period. Descriptive statistics were used to summarize dentist characteristics. Multivariable logistic regression was performed to examine associations between demographic factors and MSD-related sick leave, with adjusted odds ratios (AORs) and 95% confidence intervals (CIs).

**Results:**

Overall, 42.49% of dentists (*n* = 1,162 / 2,735) experienced at least one MSD-related sick leave episode during the study period. In adjusted analyses, dentists aged 30–39 years had higher odds of MSD-related sick leave (AOR = 1.82, 95% CI: 1.52–2.16), while those aged ≥ 50 years had lower odds (AOR = 0.66, 95% CI: 0.46–0.96), compared with those aged < 30 years. Female dentists had higher odds of MSD-related sick leave than males (AOR = 1.46, 95% CI: 1.24–1.71). Significant regional variations were also observed.

**Conclusion:**

A substantial proportion of dentists in Kuwait experienced MSD-related sick leave during the study period, particularly females and dentists in early- to mid-career stages. These findings support the implementation of early preventive ergonomic and occupational health interventions aimed at reducing MSD-related sick leave and improving workforce well-being among dentists.

**Supplementary Information:**

The online version contains supplementary material available at 10.1186/s12889-026-28153-y.

## Introduction

Musculoskeletal disorders (MSDs) are among the most common health conditions worldwide and remain a leading cause of disability and years lived with disability globally, contributing substantially to the overall burden of disease [1]. According to the Global Burden of Disease (GBD) 2021Study, musculoskeletal conditions affect more than 1.6 billion people worldwide, representing a major public health challenge across diverse populations and healthcare systems [1]. These conditions contribute substantially to pain, reduced productivity, and sickness absenteeism, resulting in considerable occupational and economic consequences worldwide [1, 2].

Dental professionals are particularly vulnerable to MSDs because dental practice involves prolonged static postures, repetitive fine motor movements, awkward body positioning, and prolonged sitting during patient care, all of which place substantial strain on the neck, shoulders, and back [3–11]. Despite advances in ergonomic equipment and training, work-related MSDs remain highly prevalent in dentistry, with a pooled global prevalence of 78.4% [12]. Consequently, MSDs are recognized as a major occupational health concern among dental professionals [13].

A substantial body of evidence demonstrates a high prevalence of MSDs among dentists across diverse settings, often affecting a majority of practitioners [8, 14–21]. Systematic reviews and meta-analyses consistently identify the neck, lower back, and shoulders as the most commonly affected anatomical regions among dental professionals [12, 13, 22]. In addition, demographic and individual factors, including age, gender, obesity, and cumulative years of practice, have also been associated with an increased risk of MSDs [12, 23–26]. Importantly, MSDs have also been associated with reduced productivity and increased absenteeism [27–30]. Recent evidence from Middle Eastern countries further demonstrated that work-related MSDs adversely affected occupational performance, concentration, and work absenteeism among dental students and dental professionals [28, 31].

Although regional studies from Gulf and Middle Eastern countries consistently report a high burden of MSDs among dentists and healthcare workers, most rely on self-reported cross-sectional designs, which may introduce recall bias, limits causal inference, and reduce generalizability, underscoring the need for objective, population-level evidence [14, 16, 23, 27, 32, 33]. Evidence from Kuwait remains limited, as existing local studies rely primarily on self-reported questionnaire-based methodologies rather than objective administrative healthcare data [28]. Furthermore, there is limited evidence using physician-certified administrative sick leave records to assess the workforce impact of MSDs among dentists, including MSD-related sickness absenteeism.

Therefore, this study aimed to estimate the prevalence of dentists experiencing at least one MSD-related sick leave episode in Kuwait and to examine associated demographic factors using Ministry of Health (MOH) administrative data. To our knowledge, this is the first nationwide study in Kuwait to investigate MSD-related sick leave among dentists using administrative sick leave records. The findings provides a population-level evidence on MSD-related sickness absenteeism and associated demographic factors, which may help inform targeted ergonomic and occupational health interventions.

## Methods

A nationwide retrospective study was conducted using administrative physician-certified sick leave records from dentists employed in Kuwait MOH facilities between 1 January 2022 and 6 October 2023. The baseline date was selected because it represented the earliest complete calendar period with standardized sick leave records available in the MOH administrative system for this study. The end date was selected to precede the implementation of electronic self-certified sick leave on 7 October 2023, after which certain sick leave certificates could be issued electronically without requiring physician-documented diagnoses [34].

Sick leave records in the administrative dataset originated from physician-certified certificates issued during clinical visits and recorded in the MOH information system by authorized clinical staff. The dataset included dentists’ demographic characteristics (age, gender, nationality, and place of residence) and sick leave information (episode date, duration, and recorded diagnosis text). Dentists employed in MOH facilities in Kuwait provide services across approximately 90 primary care and specialized dental clinics throughout all governorates, including preventive, restorative, endodontic, periodontal, orthodontic, and surgical care [35]. The MOH is the largest public healthcare provider in Kuwait and employs dentists with diverse demographic and professional backgrounds.

The source dataset included records for 2,741 dentists, representing 97.2% of the total MOH dental workforce (2,819 dentists) reported in the MOH Annual Health Report 2022 [35]. Considering that the total number of dentists in both the public and private sectors in Kuwait was 3,829 in 2022, the study population represented approximately 71.4% of Kuwait’s overall dental workforce. After excluding records with missing key demographic information (*n* = 6; 0.2%), the final analytic sample comprised 2,735 dentists. All analyses were based on complete-case data, with no missing values in variables included in the descriptive or regression analyses. All records were fully de-identified prior to analysis.

Diagnostic text entries were cleaned and standardized (e.g., converted to lowercase and trimmed) prior to classification. MSD-related sick leave episodes were identified using a predefined keyword-based algorithm applied to free-text diagnostic fields. The algorithm incorporated both full diagnostic terms (e.g., back pain, muscle, joint, sprain, strain) and root terms (e.g., musc, arth, lumb, spas) to capture variations in terminology, abbreviations, and spelling inconsistencies. The keyword list included both English and Arabic terms to account for bilingual clinical documentation and is provided in Supplementary Table S1. To improve specificity, records containing terms indicative of non-musculoskeletal conditions (e.g., upper respiratory tract infections) were excluded using a predefined exclusion list (Supplementary Table S2). The algorithm was reviewed by two public health researchers, including a dentist with clinical expertise, to improve content validity. The algorithm also underwent pilot testing and iterative refinement through review of diagnostic text entries, including the addition of root terms, abbreviations, spelling variations, and bilingual terminology to improve case detection and classification specificity.

The primary outcome was a binary indicator of MSD-related sick leave, defined as having at least one MSD-related sick leave episode during the study period. Analyses were conducted at the dentist level by aggregating individual sick leave records so that each dentist contributed only one observation to the regression model. Prevalence was defined as the proportion of dentists with at least one MSD-related sick leave episode during the study period.

Descriptive statistics were used to summarize dentist characteristics and sick leave measures. Categorical variables were presented as frequencies and percentages. The prevalence of dentists with ≥ 1 MSD-related sick leave episode was calculated overall and stratified by demographic characteristics. Descriptive frequencies and prevalence estimates were presented to facilitate comparison across subgroups.

Furthermore, to quantify the contribution of MSDs to overall sickness absenteeism, the total number of sick leave episodes and cumulative sick leave days was aggregated across all dentists. The proportions of MSD-related sick leave episodes and MSD-related sick leave days were calculated relative to all sick leave episodes and total sick leave days. In addition, among dentists with at least one MSD-related sick leave episode, cumulative MSD-related sick leave days was summarized using the median and interquartile range (IQR). These descriptive findings are summarized in the Results section.

Multivariable logistic regression was used to examine demographic factors associated with MSD-related sick leave. Covariates included age, gender, nationality, and place of residence. These variables were selected a priori based on their availability in the administrative dataset and prior literature demonstrating potential associations between demographic characteristics, MSDs, and occupational absenteeism among dental professionals [13, 15, 23, 28]. Age and gender have been consistently identified as important correlates of MSDs among dentists [12, 13, 23], while nationality and place of residence were included to account for potential sociodemographic and regional variations in healthcare workforce characteristics and work-related exposures.

Reference categories were defined a priori as dentists aged < 30 years, males, Kuwaiti nationals, and residents of Ahmadi governorate. Crude odds ratios (ORs) with 95% confidence intervals (CIs) were initially estimated using univariable logistic regression models, followed by multivariable logistic regression to estimate adjusted odds ratios (AORs) and 95% CIs. Multicollinearity was assessed using variance inflation factors (VIFs) based on an equivalent linear regression model with the same covariates. All VIF values were below 10 (mean VIF = 2.79), indicating no evidence of problematic multicollinearity. Model fit was assessed using the Hosmer–Lemeshow goodness-of-fit test. Statistical significance was defined as a two-sided p-value < 0.05. All analyses were performed using Stata version 14.2 (StataCorp, College Station, TX, USA).

## Results

A total of 2,735 dentists were included in the analysis. Table 1 presents the distribution of dentists by demographic characteristics and the subgroup-specific prevalence of ≥ 1 MSD-related sick leave episode. The largest proportion of dentists were aged < 30 years (44.79%), followed by those aged 30–39 years (34.77%), while only 7.75% were aged ≥ 50 years. Males accounted for 54.00% of the study population. The majority of dentists were Kuwaiti nationals (76.38%). With respect to place of residence, the highest proportions were observed in Hawalli (35.54%) and the Capital (Al Asimah) (25.45%).

**Table 1:** Distribution of dentists and prevalence of ≥1 MSD-related sick leave episode by demographic characteristics, Kuwait (N = 2,735)

Variable	Total dentists, *n* (%)	Dentists with ≥ 1 MSD-related sick leave episode, *n*	Prevalence within subgroup, %
Total	2735 (100.00)	1162	42.49
Age group (years)			
< 30	1225 (44.79)	474	38.69
30–39	951 (34.77)	496	52.16
40–49	347 (12.69)	133	38.33
≥ 50	212 (7.75)	59	27.83
Gender			
Male	1477 (54.00)	566	38.32
Female	1258 (46.00)	596	47.38
Nationality			
Kuwaiti	2089 (76.38)	905	43.32
Non-Kuwaiti	646 (23.62)	257	39.78
Place of residence (Governorate)			
Capital (Al Asimah)	696 (25.45)	315	45.26
Hawalli	972 (35.54)	416	42.80
Farwaniya	335 (12.25)	134	40.00
Ahmadi	116 (4.24)	37	31.90
Jahra	358 (13.09)	156	43.58
Mubarak Al-Kabeer	258 (9.43)	104	40.31

N: number of dentists; Note: MSD-related sick leave was defined as ≥1 physician-certified sick leave episode with an MSD-related diagnosis during the study period. Prevalence was calculated as the percentage of dentists with ≥1 MSD-related sick leave episode within each demographic subgroup

Overall, 1,162 dentists (42.49%) had at least one MSD-related sick leave episode during the study period. The prevalence of MSD-related sick leave varied across demographic groups (Table 1). By age group, the highest prevalence was observed among dentists aged 30–39 years (52.16%), followed by those aged < 30 years (38.69%) and 40–49 years (38.33%), while the lowest prevalence was reported among dentists aged ≥ 50 years (27.83%). Female dentists had a higher prevalence compared with males (47.4% vs. 38.3%).

In addition, MSD-related sick leave contributed notably to the overall burden of sickness absenteeism among dentists. Overall, 21.4% of all sick leave episodes and 21.2% of total sick leave days were attributable to MSD-related conditions. Among dentists with ≥ 1 MSD-related sick leave episode, the median cumulative number of MSD-related sick leave days during the study period was 3 days (IQR: 2–6 days).

Results of the multivariable logistic regression analysis are presented in Table 2. After adjustment for covariates, age, gender, and place of residence were significantly associated with MSD-related sick leave. Compared with dentists aged < 30 years, those aged 30–39 years had higher odds of MSD-related sick leave (AOR = 1.82, 95% CI: 1.52–2.16, *p* < 0.001), whereas dentists aged ≥ 50 years had lower odds (AOR = 0.66, 95% CI: 0.46–0.96, *p* = 0.029). No statistically significant difference was observed for the 40–49 age group.

**Table 2:** Crude and adjusted logistic regression analysis of demographic factors associated with MSD-related sick leave among dentists in Kuwait (*N* = 2,735)

Variable	Crude OR (95% CI)	*p*-value	Adjusted OR (95% CI)	*p*-value
Age group (years)				
< 30	Reference	—	Reference	—
30–39	1.73 (1.45–2.05)	< 0.001 *	1.82 (1.52–2.16)	< 0.001 *
40–49	0.98 (0.77–1.26)	0.902	1.03 (0.80–1.34)	0.794
≥ 50	0.61 (0.44–0.84)	0.003 *	0.66 (0.46–0.96)	0.029 *
Gender				
Male	Reference	—	Reference	—
Female	1.45 (1.24–1.69)	< 0.001 *	1.46 (1.24–1.71)	< 0.001 *
Nationality				
Kuwaiti	Reference	—	Reference	—
Non-Kuwaiti	0.86 (0.72–1.03)	0.112	1.05 (0.85–1.30)	0.656
Place of residence (Governorate)				
Ahmadi	Reference	—	Reference	—
Capital (Al Asimah)	1.77 (1.16–2.68)	0.008 *	1.62 (1.05–2.49)	0.028 *
Farwaniya	1.42 (0.91–2.23)	0.122	1.54 (0.97–2.43)	0.064
Hawalli	1.60 (1.06–2.41)	0.025 *	1.57 (1.03–2.39)	0.035 *
Jahra	1.65 (1.06–2.57)	0.027 *	1.84 (1.17–2.90)	0.008 *
Mubarak Al-Kabeer	1.44 (0.91–2.29)	0.121	1.29 (0.80–2.07)	0.293

OR: odds ratio; CI: confidence interval; *: statistically significant at *p* < 0.05. Crude ORs were estimated using univariable logistic regression models, whereas adjusted ORs were estimated using a multivariable logistic regression model including all variables shown in the table

Female dentists had higher odds of MSD-related sick leave compared with males (AOR = 1.46, 95% CI: 1.24–1.71, *p* < 0.001). Nationality was not significantly associated with MSD-related sick leave. Regarding place of residence, dentists residing in the Capital (Al Asimah) (AOR = 1.62, 95% CI: 1.05–2.49, *p* = 0.028), Hawalli (AOR = 1.57, 95% CI: 1.03–2.39, *p* = 0.035), and Jahra (AOR = 1.84, 95% CI: 1.17–2.90, *p* = 0.008) had higher odds compared with those residing in Ahmadi. No statistically significant associations were observed for Farwaniya or Mubarak Al-Kabeer. The logistic regression model demonstrated adequate goodness-of-fit based on the Hosmer–Lemeshow test (*p* = 0.255).

## Discussion

This study examined the prevalence of MSD-related sick leave among dentists in Kuwait and identified associated demographic factors. An important occupational health concern was observed, with more than 40% of dentists experiencing at least one MSD-related sick leave episode during the study period.

While previous studies have largely focused on self-reported symptom prevalence rather than functional outcomes, this finding is consistent with the high global burden of MSDs among dental professionals [8, 13, 22]. The use of sick leave as an outcome in the present study provides additional insight into the occupational and economic impact of MSDs, extending beyond self-reported discomfort to measurable work impairment. This is particularly relevant in Kuwait, where recent nationwide healthcare workforce data demonstrated that sickness absenteeism represents a substantial workforce and operational burden across healthcare professions [36]. Previous research among Finnish dentists similarly demonstrated that MSD symptoms were strongly associated with reduced work ability despite relatively low sickness absence, highlighting the importance of considering both absenteeism and presenteeism in occupational health research [29].

Age was significantly associated with MSD-related sick leave, with dentists aged 30–39 years demonstrating higher odds compared with those aged < 30 years, while those aged ≥ 50 years showed lower odds. This pattern may reflect increased clinical workload, productivity expectations, and cumulative occupational exposure during early and mid-career stages, when dentists are often managing high patient volumes and prolonged clinical working hours [37]. Dentists in this age group may also experience greater physical demands associated with establishing professional careers and maintaining intensive clinical practice schedules. Similar observations have been reported by Alexopoulos et al. [38], suggesting that MSD risk does not increase linearly with age. The lower odds among older dentists may be explained by a healthy worker effect, whereby individuals with severe MSDs reduce clinical activity or exit practice. Additionally, greater clinical experience and improved ergonomic awareness over time may contribute to reduced risk, as highlighted by Finsen et al. [21]. However, few studies have reported higher prevalence of MSD’s among the older individuals as compared with the younger [23, 28]. The cumulative exposure to occupational risk factors throughout the lifetime may contribute to this observation.

Female dentists had significantly higher odds of MSD-related sick leave compared with males, consistent with previous literature. Several previous studies have similarly reported a higher burden of MSDs among female dental professionals reported similar findings [15, 23]. This disparity may be attributed to differences in anthropometry, muscle strength, and workstation design, as well as potential psychosocial and behavioral factors influencing symptom perception and healthcare-seeking behavior.

In the present study, nationality was not significantly associated with MSD-related sick leave, suggesting that occupational exposures may play a more dominant role than sociodemographic characteristics. A cross-sectional study from Italy and Portugal reported age, years of practice, working hours, lack of breaks and posture to be the most strongly associated factors for MSD’s [24]. However, variations between countries have been reported in the literature. These differences may be attributable to several factors, including differences in healthcare systems, workplace ergonomics, workload intensity, clinical practice patterns, availability of ergonomic training, reporting practices, and methodological differences between studies. These occupational exposures may vary according to dental specialty, workload intensity, clinical practice patterns, and ergonomic working conditions, which were not available in the present administrative dataset. Furthermore, many studies measure self-reported MSD symptoms, whereas our study assessed MSD-related sick leave, which likely reflects more severe or functionally limiting conditions. In addition, the current dataset did not include direct measures of workload or workplace characteristics, and these explanations remain speculative.

The overall findings are consistent with regional and international studies demonstrating that MSDs are a significant health burden for dental professionals. Evidence from the Middle East, including studies by Alzayani et al. [33], Eddhaoui et al. [27], and Kawtharani et al. [2], supports the generalizability of these findings within similar healthcare systems. Moreover, recent studies from Europe [16] and Asia [11], indicate that MSDs remain a persistent occupational health concern across diverse settings.

From a public health perspective, the frequent occurrence of MSD-related sick leave underscores the need for preventive strategies at both individual and institutional levels. Previous studies among dental professionals have demonstrated that work-related MSDs may adversely affect productivity, occupational performance, stress levels, sleep quality, and overall quality of life [31]. Ergonomic interventions, including posture training, use of four-hand dentistry, use of micro loupes, and optimization of workstation design, have been recommended to mitigate MSD risk [2, 39, 40]. However, evidence regarding their effectiveness remains variable, as reported in the Cochrane review by Mulimani et al. [41]. Broader organizational approaches, such as workload regulation, scheduled breaks, and integration of ergonomics into dental education and continuing professional development, may provide more sustainable benefits. Change management strategies to promote ergonomic practices, as discussed by Anshasi et al. [42], may further enhance implementation.

This study has several strengths, including a focus on MSD-related sick leave as a clinically relevant outcome and the adjustment for multiple covariates in the analysis. Additional strengths include the use of a nationwide administrative dataset covering all MOH dental facilities, a large sample size, and reliance on physician-certified diagnoses, which reduces recall bias commonly observed in self-reported studies.

Nevertheless, several limitations should be considered. First, the retrospective observational design precludes causal inference between demographic factors and MSD-related sick leave. Second, the lack of information on employment dates (e.g., hire and exit dates) limited the ability to confirm active workforce status throughout the study period, which may have affected prevalence estimates. Third, the use of administrative diagnostic text may have introduced misclassification bias, as MSD-related cases were identified using a keyword-based algorithm rather than standardized clinical coding. Fourth, the regression model included a limited number of independent variables due to the constraints of the administrative dataset. Important occupational and individual factors, including dental specialty, years of professional experience, working hours, socioeconomic status, ergonomic practices, workload characteristics, psychosocial factors, and MSD severity, were unavailable for analysis. The absence of these variables may have resulted in residual confounding and limited the ability to fully explore mechanisms associated with MSD-related sick leave. Additionally, reliance on sick leave records may have underestimated the true burden of MSDs, as some dentists experiencing musculoskeletal symptoms may have continued working without taking sick leave. Also, the study population consisted of actively practicing dentists and did not include individuals who had retired or left clinical practice because of MSDs. Consequently, a healthy worker survivor effect may have occurred, potentially resulting in an underestimation of the true burden of MSD-related occupational outcomes among dentists. Finally, as the study was restricted to MOH facilities, the findings may not be fully generalizable to dentists working in private or non-governmental sectors.

Future research should use longitudinal designs to better characterize temporal relationships and incorporate detailed occupational, ergonomic, workload, and psychosocial variables not captured in administrative data. Additionally, integrating administrative data with clinical or survey-based data sources may improve measurement accuracy and provide a more comprehensive understanding of MSD-related sick leave among dentists.

## Conclusions

In conclusion, MSD-related sick leave represents a considerable occupational health burden among dentists in Kuwait, particularly among females and dentists in early to mid-career stages. The elevated odds observed among dentists aged 30–39 years suggest that preventive ergonomic and occupational health interventions should be implemented early in professional practice to reduce cumulative occupational strain and MSD-related sick leave. These findings support the need for targeted workplace modifications, ergonomic training, and occupational health strategies aimed at improving workforce well-being among dentists. Further research is needed to better understand underlying mechanisms and evaluate the effectiveness of preventive interventions in dental settings.

## Supplementary Information

Supplementary Material 1. Supplementary Table S1: Keyword list used to identify musculoskeletal disorder (MSD)-related diagnoses from administrative sick leave records and Supplementary Table S2: Exclusion Keyword List for Non-Musculoskeletal Diagnoses.

## Data Availability

The data that support the findings of this study are not publicly available due to regulatory and institutional restrictions imposed by the Kuwait Ministry of Health. The data were accessed under authorization for the current study and cannot be shared publicly. Requests for access to the data may be directed to the corresponding author and are subject to approval by the Kuwait Ministry of Health.

## Abbreviations

MSD: Musculoskeletal disorder
MOH: Ministry of Health
GBD: Global Burden of Disease
IQR: Interquartile range
OR: Odds ratio
Odds ratio: Adjusted odds ratio
VIF: Variance inflation factor
CI: Confidence interval
HSCEC: Health Science Center Ethical Committee
IRB: Institutional Review Board
