# Supplementary material for: Prevalence and demographic factors associated with MSD-related sick leave among dentists in Kuwait: a nationwide retrospective study
Source: BMC Public Health. 2026 Jun 17;26:2451. doi: 10.1186/s12889-026-28153-y (PMC13508212; doi:10.1186/s12889-026-28153-y)
Supplement: Supplementary file 1 [file 12889_2026_28153_MOESM1_ESM.docx]

**Supplementary Table S1: Keyword list used to identify musculoskeletal disorder (MSD)-related diagnoses from administrative sick-leave records**

| **Language** | **Keyword Group** | **Keyword / Term** |
| --- | --- | --- |
| English | Symptoms & general terms | back; backache; back pain; low back; bone; bone pain; muscle; muscular; cramp; spasm; joint; arthritis |
|  | Anatomical regions | knee; shoulder; neck; lumbar |
|  | Diagnoses & conditions | sprain; strain; disc; tendon; ligament; sciatica |
|  | Extremities | foot pain; hand pain |
|  | Root terms (partial matches) | musc; spas; arth; lumb |
| Arabic | Symptoms & general terms | الظهر؛ الضهر؛ ألم الظهر؛ وجع الظهر؛ أسفل الظهر |
|  | Musculoskeletal structures | العظم؛ ألم العظم؛ العضلة؛ تشنج عضلي؛ المفصل؛ التهاب المفاصل |
|  | Anatomical regions | الركبة؛ الكتف؛ الرقبة؛ الفقرات القطنية |
|  | Diagnoses & conditions | التواء؛ إجهاد؛ القرص؛ الوتر؛ الرباط؛ عرق النسا |
|  | Extremities | ألم القدم؛ ألم اليد |

**Supplementary Table S2: Exclusion Keyword List for Non-Musculoskeletal Diagnoses.**

| **Category** | **Keywords / Terms Included** |
| --- | --- |
| Respiratory infections | urti, upper respiratory |
| Viral infections | flu, influenza, cold |
| Respiratory symptoms | cough, fever |
| Upper airway infections | pharyngitis, tonsillitis, laryngitis |
| Lower respiratory infections | bronchitis |
| Sinus-related conditions | sinusitis |
